# Association Between Gestational Hypertension and Risk of Cardiovascular Disease Among 617 589 Norwegian Women

**DOI:** 10.1161/JAHA.117.008337

**Published:** 2018-05-13

**Authors:** Hilde Kristin Refvik Riise, Gerhard Sulo, Grethe S. Tell, Jannicke Igland, Ottar Nygård, Ann‐Charlotte Iversen, Anne Kjersti Daltveit

**Affiliations:** ^1^ Department of Global Public Health and Primary Care University of Bergen Norway; ^2^ Division of Mental and Physical Health Norwegian Institute of Public Health Bergen Norway; ^3^ Department of Heart Disease Haukeland University Hospital Bergen Norway; ^4^ KG Jebsen Center for Diabetes Research Department of Clinical Science University of Bergen Norway; ^5^ Centre of Molecular Inflammation Research and Department of Clinical and Molecular Medicine Norwegian University of Science and Technology (NTNU) Trondheim Norway; ^6^ Department of Health Registries Norwegian Institute of Public Health Bergen Norway

**Keywords:** cardiovascular disease, fetal growth restriction, gestational hypertension, preeclampsia/pregnancy, preterm delivery, Cardiovascular Disease, Epidemiology, Pregnancy, Women

## Abstract

**Background:**

Preeclampsia and gestational hypertension (GH) are the most common hypertensive pregnancy disorders. Preeclampsia has been linked to increased risk of cardiovascular disease (CVD), but a similar association for GH has not been established. We aimed to determine the association between GH and subsequent CVD, and explore the additional role of small‐for‐gestational‐age infants, preterm delivery, and parity.

**Methods and Results:**

Data from the Medical Birth Registry of Norway were linked to the Cardiovascular Disease in Norway project and the Norwegian Cause of Death Registry. Hazard ratios and 95% confidence intervals were computed using Cox proportional hazard regression, comparing women with and without GH during their first and/or second pregnancy. We included all women with a first delivery from 1980 through 2009 (n=617 589) and followed them for a median of 14.3 (quartile 1–quartile 3: 6.9–21.5) years. Women with GH in the first pregnancy had 1.8‐fold (95% confidence interval, 1.7–2.0) higher risk of subsequent CVD compared with women without any hypertensive pregnancy disorder. When GH occurred in combination with small‐for‐gestational‐age infants and/or preterm delivery, the hazard ratio was 2.6 (95% confidence interval, 2.3–3.0). When women with GH were compared with women with preeclampsia, the risk of CVD was comparable when the pregnancy complications occurred in either the first or second pregnancy but was significantly higher for preeclampsia without complications when the disorder occurred in both pregnancies.

**Conclusions:**

GH was associated with increased risk of subsequent CVD, and the highest risk was observed when GH was combined with small‐for‐gestational‐age infants and/or preterm delivery.

**Clinical Perspective**

**What Is New?**

- Gestational hypertension increases the risk of maternal cardiovascular disease later in life, and the risk is further elevated with the presence of a small‐for‐gestational‐age infant and/or preterm delivery.

**What Are the Clinical Implications?**

- Gestational hypertension should be considered in addition to preeclampsia when assessing a woman's future risk of cardiovascular disease.

## Introduction

Hypertensive pregnancy disorders are associated with subsequent maternal cardiovascular disease (CVD) morbidity and mortality^1^, ^2^, ^3^, ^4^, ^5^ and pregnancy is considered a vascular stress test for later CVD risk. Hypertensive pregnancy disorders complicate around 5% to 10% of pregnancies worldwide,^6^ and gestational hypertension (GH) and preeclampsia account for most of these cases. GH is most common and affects 5% to 8% of healthy women,^6^, ^7^, ^8^, ^9^, ^10^ while preeclampsia complicates 2% to 7% of all pregnancies.^11^ There are important similarities and differences in the causes and pathophysiology of GH and preeclampsia.^12^, ^13^, ^14^ Risk factors for both conditions include overweight, obesity, and diabetes mellitus.^13^, ^14^ Only preeclampsia occurs more often in first pregnancies.^15^ Differences in the pathophysiology between the 2 disorders are indicated by higher serum cytokine levels in the early phase of GH and stronger involvement of placental dysfunction in preeclampsia.^12^, ^16^, ^17^, ^18^

An association between hypertensive pregnancy disorders and other adverse pregnancy outcomes such as small‐for‐gestational‐age (SGA) infants and preterm delivery is of clinical importance for preventing serious disease in both mother and child.^12^ There is a well‐established association between preeclampsia, alone or in combination with SGA infants and/or preterm delivery, and subsequent risk of maternal CVD.^1^, ^2^, ^3^, ^5^, ^19^, ^20^, ^21^, ^22^, ^23^ In a study from 2016, we showed a doubled risk of CVD when preeclampsia occurred alone and a 4‐fold risk when preeclampsia occurred in combination with SGA and/or preterm delivery.^2^ A possible relationship between GH and risk of maternal CVD has been studied to a much smaller extent. While several studies have reported a significant association between GH and subsequent CVD,^5^, ^19^, ^20^, ^23^, ^24^, ^25^, ^26^, ^27^, ^28^, ^29^, ^30^ only 4 of these had a comparable cohort size and a similar follow‐up time compared with our study.^19^, ^20^, ^23^, ^29^ None of these studies looked at the combined effects of GH, SGA infant, preterm delivery, and parity as predictors of subsequent CVD.

This study was undertaken to explore the role of GH on subsequent CVD events, accounting for the additional role of SGA and/or preterm delivery as well as the potential modifying effect of parity in this association. The GH associated risk was further compared with that of preeclampsia. We examined these associations in a prospective population‐based cohort study by linking data from the Medical Birth Registry of Norway (MBRN) with the Norwegian Cause of Death Registry and the Cardiovascular Disease in Norway (CVDNOR) project, covering up to 29 years of follow‐up.

## Methods

The data and study material will not be made available to other researchers for purposes of reproducing the results or replicating the procedure by reason of ethical and data protective legislation.

### Study Design and Study Population

The MBRN is a national registry established in 1967, based on compulsory notification of live births and stillbirths. The registry contains information on all pregnancies beyond 16 weeks of gestation, including maternal characteristics and medical history, as well as pregnancy complications.^31^ To follow women with regard to later development of CVD, we linked the MBRN to the CVDNOR project (http://www.cvdnor.no). Further linkage with the Norwegian Cause of Death Registry, Statistics Norway, and the National Population Registry provided us with information of cause and date of death (1980–2009), sociodemographic status, and date of emigration, respectively.

**Figure 1:**
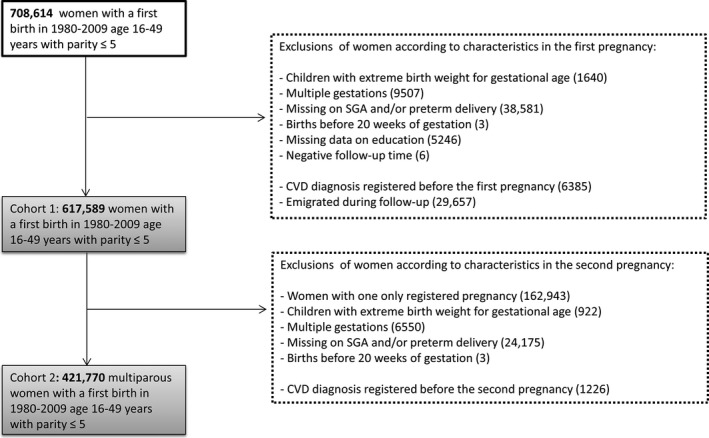
Flowchart describing the study population of 617 589 Norwegian women followed from 1980 through 2009, including exclusion criteria. CVD indicates cardiovascular disease; preterm delivery, <37 weeks of gestation; SGA, small‐for‐gestational‐age (<10th percentile).

A total of 708 614 women were recorded in the MBRN in the study period (1980–2009). From this population we defined 2 cohorts. Cohort 1 was defined as all women aged 16 to 49 years who had their first delivery during 1980–2009. Only information from the first pregnancy is included in the analyses and time of delivery represents start (baseline) of the follow‐up period. A total of 29 657 (4.2%) women emigrated during the follow‐up period and were excluded from the study, leaving 678 957 eligible women. Exclusions were defined according to the woman's first pregnancy and CVD history. Based on information in the MBRN or the CVDNOR project, 6385 women with existing CVD (*International Classification of Diseases [ICD], Tenth Revision*: I00–I99 and corresponding codes for *ICD‐8* and *ICD‐9*) at baseline were excluded. We further excluded women with babies who had a *z* score outside (−4, +4) of birthweight by gestational week (n=1640), missing information on SGA and/or preterm delivery (n=38 581), and multiple gestation (n=9507). Women with births before 20 weeks of gestation (n=3) were excluded, since hypertensive pregnancy disorders are diagnosed after 20 weeks of gestation. In addition, we excluded women with missing information on education (n=5246) and women with negative follow‐up time, likely attributable to an erroneous date of death (n=6). The final cohort 1 included 617 589 women (Figure 1).

Cohort 2 was defined as women in cohort 1 with at least 2 pregnancies (n=454 646), and follow‐up started at the second delivery. Information from the first and second pregnancies was included and further exclusions were based on characteristics in the second pregnancy: delivery of a baby with a *z* score outside (−4, +4) of birthweight by gestational week (n=922), missing information on SGA and/or preterm delivery (n=24 175), multiple gestation (n=6550), and births before 20 weeks of gestation (n=3). We also excluded 1226 women with a CVD diagnosis before the second pregnancy. The final cohort 2 included 421 770 women (Figure 1).

Two different sets of additional analyses were performed. First, we analyzed all women using information on hypertensive pregnancy disorders in any of the women's pregnancies (up to 5 pregnancies). After exclusions according to information in all of the women's pregnancies we were left with 577 185 women for these analyses (for details see Table S1). Second, further analyses were performed on women with ≥2 pregnancies (n=413 701).

The study was approved by the Regional Committee for Medical and Health Research Ethics (2014/1047). Individual informed consent was waived because data were collected from national mandatory registries.

### Study Exposure and End Points

The MBRN defines GH as hypertension occurring after 20 weeks of gestation (systolic blood pressure [BP] ≥140 mm Hg and/or diastolic BP ≥90 mm Hg, or an increase >15 mm Hg from BP measured before gestational week 20). A preeclampsia diagnosis requires the additional presence of proteinuria (≥0.3 g in 24‐hour urine or a ≥1‐point increase on dipstick) and also includes eclampsia and HELLP (hemolysis, elevated liver enzymes, low platelet count) syndrome. Preterm delivery was defined as delivery less than 37 weeks of gestation and SGA was defined as fetal growth below the 10th percentile of Norwegian birth weight curves.^32^

The primary end point was defined as CVD (*ICD‐9* codes 390–459; *ICD‐10* codes I00–I99 [except I84]). Secondary end points were coronary heart disease (CHD) (*ICD‐9* codes 410–414; *ICD‐10* codes I20–I25) and cerebrovascular disease (*ICD‐9* codes 430–438, *ICD‐10* codes I60–I69).

### Statistical Methods

Descriptive characteristics are reported as means, SDs, medians, interquartile range, and percentages. Time‐to‐event analyses were conducted using Cox proportional hazard regression to estimate hazard ratios (HRs) and 95% confidence intervals (CIs) for each study outcome, comparing women with and without GH or preeclampsia. Proportional hazard assumption was checked by inspecting log‐(log) survival plots for all relevant variables. The level of significance was defined as *P*<0.05 in all analyses (2‐sided). In all models we considered educational level, marital status, and birth year of first child as potential confounders. Educational level was classified into 3 categories: basic (compulsory) education, upper secondary education (high school or vocational school), and tertiary education (college or university). Information on smoking, body mass index, and alcohol consumption was not available.

In cohort 1, we analyzed the risk of CVD in women with or without GH in their first pregnancy. Follow‐up time was calculated as the difference between the mother's age at the date of hospital admission, death, or December 31, 2009 (whichever occurred first), and age at first delivery. Women were stratified into: (1) no GH, no preeclampsia, or no SGA or preterm delivery; (2) GH only; (3) SGA only; (4) preterm delivery only; and (5) GH with SGA and/or preterm delivery.

In cohort 2, follow‐up time was calculated as the difference between the mother's age at the date of hospital admission, death, or December 31, 2009 (whichever occurred first), and age at second delivery. Analyses were stratified by first and/or second pregnancy, and we assessed whether GH alone or combined with SGA and/or preterm delivery differed in CVD risk according to parity. In separate analyses we directly compared the obtained HRs and *P* values for risk of CVD between women with GH and women with preeclampsia, with or without the presence of SGA infants and/or preterm delivery.

In the first sets of additional analyses (n=577 185), looking at GH in any pregnancy, we introduced GH as a time‐dependent covariate in order to avoid classifying the mother as exposed while still unexposed before occurrence of her first pregnancy with GH. In addition to educational level, marital status, and birth year of first child, we also adjusted for parity and preeclampsia as time‐dependent covariates. By adjusting for preeclampsia, we estimated the part of the effect of GH on risk of CVD that was not influenced by a subsequent preeclamptic pregnancy in the same woman. In the second set of additional analyses (n=413 701), we examined the risk of CVD in women with both GH and preeclampsia. Hypertensive pregnancy disorder was introduced as a time‐dependent covariate, and women were stratified into: (1) no GH, no preeclampsia; (2) GH; (3) preeclampsia; or (4) both GH and preeclampsia. The women were followed from age at their first delivery in both sets of additional analyses.

## Results

### Characteristics of the Study Population

Characteristics according to hypertensive pregnancy disorder status in the first pregnancy (cohort 1) are summarized in Table 1. A total of 41 434 (6.5%) women had a hypertensive pregnancy disorder in the first pregnancy, distributed as 11 600 (28.0%) women with GH and 29 834 (72.0%) women with preeclampsia.

**Table 1:** Distribution of 617 589 Norwegian Women With a First Pregnancy During 1980–2009 According to Hypertensive Pregnancy Disorders in the First Pregnancy (Cohort 1)

	No Hypertensive Pregnancy Disorder (n=576 155)^a^	Hypertensive Pregnancy Disorder (n=41 434)			
		GH (n=11 600)	*P* Value^b^	Preeclampsia (n=29 834)	*P* Value^c^
Mother's age, mean (SD), y	26.3 (5.0)	27.4 (5.0)	<0.001	26.6 (5.0)	<0.001
Education level			<0.001		<0.001
Basic education, No. (%)	155 363 (27.0)	2882 (24.8)		8123 (27.2)	
Secondary education, No. (%)	173 290 (30.0)	3342 (28.8)		9345 (31.3)	
Tertiary education, No. (%)	247 502 (43.0)	5376 (46.3)		12 366 (41.5)	
Marital status			<0.001		<0.001
Married/cohabitants, No. (%)	482 982 (83.8)	10 200 (87.9)		25 523 (85.5)	
Other, No. (%)	93 173 (16.2)	1400 (12.1)		4311 (14.5)	
Diabetes mellitus, No. (%)^d^	2063 (0.4)	92 (0.8)	<0.001	497 (1.7)	<0.001
Maternal complications			<0.001		0.001
Placental abruption, No. (%)	2764 (0.5)	93 (0.8)		355 (1.2)	
Infant characteristics					
Preterm delivery, No. (%)	31 206 (5.4)	811 (7.0)	<0.001	6154 (20.6)	<0.001
SGA infant, No. (%)	67 968 (11.8)	1913 (16.5)	<0.001	7052 (23.6)	<0.001
Stillbirth, No. (%)	3339 (0.6)	91 (0.8)	0.004	220 (0.7)	0.618
Mode of delivery			<0.001		<0.001
Spontaneous, No. (%)	486 159 (84.4)	7467 (64.4)		11 747 (39.4)	
Induced, No. (%)	70 563 (12.3)	3541 (30.5)		14 353 (48.1)	
Cesarean, No. (%)	19 433 (3.4)	592 (5.1)		3734 (12.5)	

Preterm delivery indicates <37 weeks of gestation; SGA, small‐for‐gestational‐age (<10th percentile).

a No hypertensive pregnancy disorder indicates no gestational hypertension (GH) or preeclampsia.

b *P* value compares differences among women with no hypertensive pregnancy disorder vs women with GH. Chi‐square test for categorical data and *t* test for continuous data.

c *P* value compares differences among women with GH vs women with preeclampsia. Chi‐square test for categorical data and *t* test for continuous data.

d Diabetes mellitus (defined as type 1, type 2, or unspecified) diagnosed before the first pregnancy.

Women with GH were more likely than women without hypertensive pregnancy disorders to be married/cohabitants and to have higher education. The occurrence of diabetes mellitus, placental abruption, preterm deliveries, SGA infants, and stillbirths was higher among women with GH compared with women without hypertensive pregnancy disorders. Women with GH had a higher proportion of cesarean section (*P*<0.001) compared with women without hypertensive pregnancy disorders.

Compared with women with preeclampsia, women with GH were less likely to have diabetes mellitus, preterm delivery, and SGA infants. The proportion of cesarean sections was significantly higher among women with preeclampsia compared with women with GH (12.5% versus 5.1%, respectively; *P*<0.001).

During a median of 14.3 (quartile 1–quartile 3: 6.9–21.5) years, follow‐up after first delivery, 21 819 (3.5%) women developed CVD, of whom 2885 (13.2%) had CHD and 2657 (12.2%) had cerebrovascular disease (Table 2). Among women without hypertensive pregnancy disorders, there were 19 111 (3.3%) occurrences of CVD, 2517 (0.4%) CHD events, and 2383 (0.4%) cerebrovascular events. These occurrences were all significantly higher (*P*<0.001) in women with GH in their first pregnancy, with 758 (6.5%), 99 (0.9%), and 69 (0.6%) events, respectively.

**Table 2:** Distribution of Morbidity and Mortality of CVD in 617 589 Norwegian Women (Median Follow‐Up of 14.3 Years) With a First Pregnancy in 1980–2009 According to Hypertensive Pregnancy Disorders in the First Pregnancy (Cohort 1)

	No Hypertensive Pregnancy Disorder (n=576 155)^a^	Hypertensive Pregnancy Disorder (n=41 434)			
		GH (n=11 600)	*P* Value^b^	Preeclampsia (n=29 209)	*P* Value^c^
CVD	19 111 (3.3)	758 (6.5)	<0.001	1950 (6.5)	0.995
Hospitalizations	17 628 (92.2)	715 (94.3)		1831 (93.9)	
Deaths	1438 (7.8)	43 (5.7)		119 (6.1)	
CHD	2517 (0.4)	99 (0.9)	<0.001	269 (0.9)	0.639
Hospitalizations	2272 (90.3)	92 (92.9)		230 (85.5)	
Deaths	245 (9.7)	7 (7.1)		39 (14.5)	
Cerebrovascular disease	2383 (0.4)	69 (0.6)	0.003	205 (0.7)	0.298
Hospitalizations	2054 (86.2)	59 (85.5)		180 (87.8)	
Deaths	329 (13.8)	10 (14.5)		25 (12.2)	

Values are expressed as number (percentage). CHD indicates coronary heart disease; CVD, cardiovascular disease.

a No hypertensive pregnancy disorder indicates no gestational hypertension (GH) or preeclampsia.

b *P* value compares differences among women with no hypertensive pregnancy disorder vs women with GH. Chi‐square test for categorical data and *t* test for continuous data.

c *P* value compares differences among women with GH vs women with preeclampsia. Chi‐square test for categorical data and *t* test for continuous data.

### GH in First Pregnancy and Risk of Subsequent CVD

**Figure 2:**
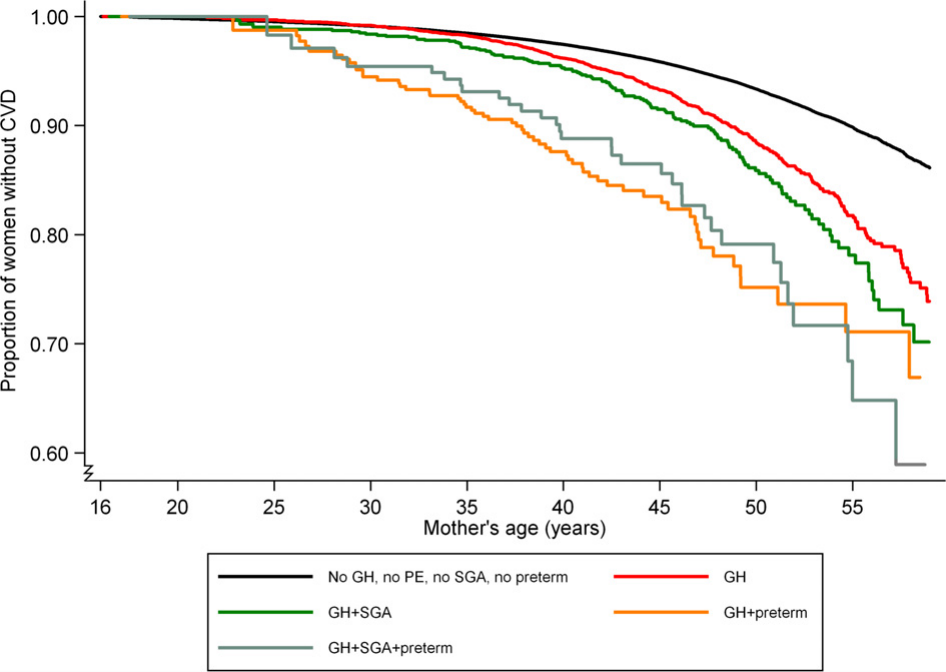
Kaplan–Meier curve of cardiovascular disease risk for 617 589 Norwegian women with ≥1 pregnancies according to a hypertensive pregnancy disorder in the first pregnancy (cohort 1). CVD indicates cardiovascular disease; GH, gestational hypertension; PE, preeclampsia; preterm delivery, <37 weeks of gestation; SGA, small‐for‐gestational‐age (<10th percentile).

The GH‐associated risks for CVD (alone or in combination with SGA and/or preterm delivery) in cohort 1 are shown in Figure 2 and Table 3. Women with GH showed higher risk of CVD compared to women without hypertensive pregnancy disorders; HR, 1.8 (95% CI, 1.7–2.0) for GH alone, increasing to 2.6 (95% CI, 2.3–3.0) when GH occurred in combination with SGA and/or preterm delivery. Women with SGA only or preterm delivery only had an increased risk of CVD with HRs of 1.1 (95% CI, 1.1–1.2) and 1.3 (95% CI, 1.2–1.3), respectively, compared with women with no hypertensive pregnancy disorders. Comparable results were found for CHD and cerebrovascular disease, although the association between GH and cerebrovascular disease was not significant (Table 3).

**Table 3:** Associations Between GH in First Pregnancy and Risk of CVD Among 617 589 Women Followed From 1980 to 2009 (Cohort 1)

	No./Events	Unadjusted HR (95% CI)	Adjusted HR (95% CI)
CVD			
No HPD, no SGA, no preterm delivery	481 500/15 182	1	1
GH only	9148/534	1.8 (1.7–2.0)	1.8 (1.7–2.0)
SGA only	63 449/2580	1.1 (1.1–1.2)	1.1 (1.1–1.2)
Preterm delivery only	26 687/1086	1.3 (1.2–1.4)	1.3 (1.2–1.3)
GH and SGA and/or preterm delivery	2452/224	2.7 (2.4–3.1)	2.6 (2.3–3.0)
CHD			
No HPD, no SGA, no preterm delivery	481 500/1863	1	1
GH only	9148/65	1.7 (1.3–2.2)	1.7 (1.3–2.1)
SGA only	63 449/464	1.6 (1.5–1.8)	1.5 (1.4–1.7)
Preterm delivery only	26 687/144	1.4 (1.2–1.7)	1.3 (1.1–1.6)
GH and SGA and/or preterm delivery	2452/34	2.9 (2.1–4.1)	2.8 (2.0–3.9)
Cerebrovascular disease			
No HPD, no SGA, no preterm delivery	481 500/1824	1	1
GH only	9148/45	1.3 (0.9–1.7)	1.3 (0.9–1.7)
SGA only	63 449/364	1.3 (1.2–1.5)	1.3 (1.2–1.4)
Preterm delivery only	26 687/152	1.5 (1.3–1.8)	1.4 (1.2–1.7)
GH and SGA and/or preterm delivery	2452/24	2.3 (1.5–3.5)	2.3 (1.5–3.4)

Cox proportional hazard regression analyses with mother's age as the time scale. Follow‐up starts at mother's age at first delivery. Category with other combinations (ie, preeclampsia alone, small‐for‐gestational‐age [<10th percentile] [SGA]+preterm delivery) not shown (n=34 353). Adjusted for educational level, marital status, and birth year of first child (all are significant in full model). CHD indicates coronary heart disease; CI, confidence interval; CVD, cardiovascular disease; GH, gestational hypertension; HPD, hypertensive pregnancy disorder; HR, hazard ratio; preterm delivery, <37 weeks of gestation.

### GH in the First and/or Second Pregnancy and Risk of Subsequent CVD

Table 4 shows results for more in‐depth analyses of GH in combination with SGA and/or preterm delivery of women in cohort 2. Among women with GH only, the risk was lowest for GH in the first pregnancy (HR, 1.7; 95% CI, 1.5–2.0), intermediate for GH in both pregnancies (HR, 1.9; 95% CI, 1.3–2.6), and highest for GH in the second pregnancy (HR, 2.4; 95% CI, 2.1–2.8). When GH occurred in conjunction with SGA and/or preterm delivery, the risk was highest for women with GH in both pregnancies (HR, 3.6; 95% CI, 2.4–5.2).

**Table 4:** GH in First and/or Second Pregnancy and Risk of CVD Among 421 770 Women With at Least 2 Pregnancies Followed From 1980 to 2009 (Cohort 2)

	No./Events	HR (95% CI)^a^
No HPD, no SGA, no preterm delivery^b^	302 623/8828	1
GH only		
First, but not second pregnancy	4901/254	1.7 (1.5–2.0)
Second, but not first pregnancy	2646/180	2.4 (2.1–2.8)
Both pregnancies	543/32	1.9 (1.3–2.6)
GH and SGA and/or preterm delivery		
First, but not second pregnancy	1139/71	2.0 (1.6–2.5)
Second, but not first pregnancy	418/39	3.0 (2.2–4.1)
Both pregnancies^c^	230/26	3.6 (2.4–5.2)

Cox proportional hazard regression analyses with mother's age as the time scale. Follow‐up starts at mother's age at second delivery. Women with preeclampsia are excluded from the analysis (n=24 026). Category with other combinations of gestational hypertension (GH)/small‐for‐gestational‐age (<10th percentile) (SGA) infant/preterm not shown (n=85 244). CI indicates confidence interval; CVD, cardiovascular disease; HPD, hypertensive pregnancy disorder; HR, hazard ratio; preterm delivery, <37 weeks of gestation.

a Adjusted for educational level, marital status, and birth year of first child (all are significant in full model).

b No GH, preeclampsia, SGA, or preterm delivery in first or second pregnancy.

c GH in both pregnancies, SGA/preterm in at least 1 pregnancy.

### Comparison of GH and Preeclampsia

In general, the risk of CVD after GH or preeclampsia appeared to be similar; however, recurrent GH had a significantly lower risk of CVD compared with recurrent preeclampsia when the disorders occurred without SGA/preterm birth (HR, 0.5; 95% CI, 0.4–0.8) (Table 5).

**Table 5:** GH or Preeclampsia in First and/or Second Pregnancy and Risk of CVD Among 421 770 Women With at Least 2 Pregnancies Followed From 1980 to 2009 (Cohort 2)

	Preeclampsia		GH	
	No./Events	HR (95% CI)	No./Events	HR (95% CI)^a^
HPD only				
First, but not second pregnancy	10 455/493	1 (reference)	4901/254	1.0 (0.8–1.1)
Second, but not first pregnancy	3855/261	1 (reference)	2646/180	1.0 (0.9–1.2)
Both pregnancies	1389/133	1 (reference)	543/32	0.5 (0.4–0.8)^b^
HPD and SGA and/or preterm delivery				
First, but not second pregnancy	4813/204	1 (reference)	1139/71	1.2 (1.0–1.6)
Second, but not first pregnancy	1693/131	1 (reference)	418/39	1.1 (0.8–1.5)
Both pregnancies^c^	1821/185	1 (reference)	230/26	0.9 (0.6–1.4)

Cox proportional hazard regression analysis with mother's age as the time scale. Follow‐up starts at mother's age at second delivery. Women without preeclampsia or gestational hypertension (GH) in the 2 first pregnancies (302 623) and women with other combinations of GH/preeclampsia/small‐for‐gestational‐age (<10th percentile) (SGA) infant/preterm delivery (ie, women with GH in the first and preeclampsia in the second or vice versa) not shown (n=85 244). CI indicates confidence interval; CVD, cardiovascular disease; HPD, hypertensive pregnancy disorder; HR, hazard ratio; preterm delivery, <37 weeks of gestation.

a Adjusted for educational level, marital status, and birth year of first child (all are significant in full model).

b Significantly different from preeclampsia without SGA/preterm in both pregnancies.

c HPD in both pregnancies, SGA infant and/or preterm delivery in at least 1 of the 2 pregnancies.

### Additional Analyses

When studying GH in any of the women's pregnancies (n=577 185), we found an HR of 2.0 (95% CI, 1.9–2.2) for the risk of subsequent maternal CVD (Table S1). After adjustment for educational level, marital status, birth year of first child, parity (time‐dependent), and preeclampsia (time‐dependent), the HR decreased to 1.9 (95% CI, 1.8–2.0). This indicates that the effect of GH on risk of CVD is not caused by a subsequent preeclamptic pregnancy and that GH independently increases the risk of subsequent maternal CVD. The same pattern was found for CHD and cerebrovascular disease. When analyzing women with ≥2 pregnancies (n=413 701), we found an HR of 3.3 (95% CI, 2.9–3.7) for women who experienced both hypertensive pregnancy disorders and risk of subsequent maternal CVD (adjusted for educational level, marital status, birth year of first child, and parity [time‐dependent]). This is higher than what we found for women who experienced only 1 of these hypertensive pregnancy disorders (GH: HR, 2.0 [95% CI, 1.8–2.2]; preeclampsia: HR, 2.1 [95% CI, 2.0–2.2]) (data not shown in Tables).

## Discussion

In this large cohort study based on nationwide health registries, we found that GH increased the risk of subsequent CVD both overall and for its major components of CHD and cerebrovascular disease. When complicated with SGA and/or preterm delivery, GH‐associated risk for CVD was further increased. The detrimental effect of GH was more pronounced when it occurred in the second compared with the first pregnancy. The risk for subsequent CVD was similar for preeclampsia and GH when complicating only 1 pregnancy, while for recurrent uncomplicated hypertensive pregnancy disorder, the adverse effect of preeclampsia was more pronounced than for GH.

### Comparison With Other Studies

Most previous studies of the association between hypertensive pregnancy disorders and future maternal CVD have either examined GH and preeclampsia combined or focused solely on preeclampsia.^1^, ^3^, ^33^, ^34^ The risk of future CVD for GH alone has been less studied, but some previous studies support our finding of a positive association between GH and cardiovascular events.^19^, ^20^, ^23^, ^24^, ^25^, ^26^, ^29^, ^30^ Four of these are large Scandinavian register‐based cohort studies with long follow‐up periods^19^, ^20^, ^23^, ^29^ that observed higher risk of CVD morbidity and mortality in women who experienced GH, with the highest CVD risk for the highest degree of severity of GH^20^ and stronger association with postpregnancy hypertension for GH than preeclampsia.^29^ Three studies^24^, ^25^, ^26^ reported that GH as well as preeclampsia increased the risk of CVD. Other studies report more inconsistent, conflicting, or negative results, with no association between GH alone and CVD for nonblacks after stratifying by race,^5^ with a positive association between GH and CVD but not for thromboembolic events (deep venous thrombosis or pulmonary embolism),^27^ and lack of positive association between GH and CVD.^28^ Compared with our study, these studies had limitations in terms of small population size and relatively short follow‐up for cardiovascular events.^25^, ^26^, ^27^, ^28^

The role of GH combined with SGA and/or preterm delivery on future CVD has been addressed in a few studies.^5^, ^20^, ^24^, ^28^ These studies support our findings, but have limitations. A Swedish study of GH or preeclampsia combined with SGA or preterm delivery found an incidence rate ratio of 2.6 for subsequent risk of ischemic heart disease; however, most analyses combined GH and preeclampsia.^20^ Two studies of the combination of maternal placental syndrome with SGA/preterm delivery found an increased risk of CVD, but without specifying the types of hypertensive pregnancy disorders.^24^, ^28^ The study presented here is the first large cohort study analyzing GH as a separate disorder combined with SGA and/or preterm delivery and we observed a strong and significant increased risk of CVD in these women.

To the best of our knowledge this is the first study to explore how parity affects the association between GH (with or without SGA and/or preterm delivery) and subsequent risk of CVD. Interestingly, we observed that whether GH occurred in the first or second pregnancy profoundly affected the risk of CVD, and that GH and preeclampsia showed comparable patterns in this respect, implying comparable effects of parity for the 2 conditions. For preeclampsia, a different cause for a group of women only experiencing the disorder in their first pregnancy is acknowledged^15^ and it has been shown that the risk for future CVD is higher when preeclampsia occurs in the second pregnancy compared with the first.^2^, ^19^ Such a parity‐specific pattern has not previously been shown for GH and our findings point to differences in severity of the maternal disorder or underlying maternal characteristics when comparing the occurrence of GH in a woman's first or second pregnancy. For recurrent hypertensive pregnancy disorder, a difference between GH and preeclampsia was apparent with higher preeclampsia‐associated CVD risk compared with GH. Together, our findings point to both shared and distinct underlying processes in these disorders. The difference between GH and preeclampsia is reflected by the highest risk for future CVD in women who experience both GH and preeclampsia and suggests that both disorders have a unique contribution to the vascular load and risk of CVD later in life.

### Strengths and Limitations

The major strengths of our study are inclusion of a large nationwide cohort consisting of more than 600 000 women and detailed follow‐up of both nonfatal and fatal CVD over a period of up to 29 years. We were able to investigate relatively young women in a homogeneous, nonselected low‐risk population. The national personal identification number allowed linking of data from several sources of information, thus improving the completeness of information and ensuring minimal loss to follow‐up. Some limitations also need to be addressed. Inclusions of deliveries from 1980 to 1993 gave up to 14 years without morbidity follow‐up. However, the majority of CVD events in women occur after age 50 years and the median age at first delivery was 26 years; thus, the number of CVD events before 1994 was expected to be low. Complete follow‐up for CVD mortality was available from 1980 through 2009, as previously described.^2^ The diagnostic validity for preeclampsia in the MBRN is high,^35^, ^36^ while the positive predictive value for GH has been found to be 68% in a validity study with data from the MBRN and the population‐based HUNT (Nord‐Trøndelag Health) study in Nord‐Trøndelag County.^37^ This study did not have the possibility to apply the newer stricter criteria for GH that requires at least 2 high BP recordings.^6^ The low prevalence of GH in our study may be a result of underreporting of the less severe cases to the MBRN, possibly making this diagnosis less reliable.^19^ Moreover, it is acknowledged that chronic hypertension is underdiagnosed in young women.^38^ Women may have their first BP measured during pregnancy and thereby receive a misdiagnosis of GH instead of chronic hypertension. Another weakness of our study is the inability to adjust for several confounders including smoking, body mass index, and alcohol consumption, attributable to missing information in the registries. We can therefore not exclude the possibility that these risk factors might play a role in the association between hypertensive pregnancy disorders and CVD.

## Conclusions

Results from our study suggest that more attention should be given to the course and consequence of GH, and that GH and preeclampsia should be addressed as separate disorders in future studies. The results related to parity and risk of future CVD point out that recognition of different subtypes of GH and preeclampsia is important for prevention and treatment of CVD and that special attention should be given to women at highest risk of future CVD. Our findings identify a new severity of GH with regard to future maternal health and underline the need for further attention toward these affected women.

## Sources of Funding

Hilde Kristin Refvik Riise, MSN, received a scholarship from “Extrastiftelsen, Nasjonalforeningen for Folkehelsen,” Oslo, Norway.

## Disclosures

None.

## Supporting information


**Table S1.** HRs and 95% CIs Using Time‐Dependent Covariates for Associations Between GH in at Least 1 Pregnancy During 1980–2009 and Risk of CVD Among 577 185 Women

## References

[1] Brown MC , Best KE , Pearce MS , Waugh J , Robson SC , Bell R . Cardiovascular disease risk in women with pre‐eclampsia: systematic review and meta‐analysis. Eur J Epidemiol. 2013;28:1–19. 23397514 10.1007/s10654-013-9762-6

[2] Riise HK , Sulo G , Tell GS , Igland J , Nygard O , Vollset SE , Iversen AC , Austgulen R , Daltveit AK . Incident coronary heart disease after preeclampsia: role of reduced fetal growth, preterm delivery, and parity. J Am Heart Assoc. 2017;6:e004158 DOI: 10.1161/JAHA.116.004158. 28264858 10.1161/JAHA.116.004158 PMC5523993

[3] Skjaerven R , Wilcox AJ , Klungsoyr K , Irgens LM , Vikse BE , Vatten LJ , Lie RT . Cardiovascular mortality after pre‐eclampsia in one child mothers: prospective, population based cohort study. BMJ. 2012;345:e7677. 23186909 10.1136/bmj.e7677 PMC3508198

[4] Chen CW , Jaffe IZ , Karumanchi SA . Pre‐eclampsia and cardiovascular disease. Cardiovasc Res. 2014;101:579–586. 24532051 10.1093/cvr/cvu018 PMC3941598

[5] Cirillo PM , Cohn BA . Pregnancy complications and cardiovascular disease death: 50‐year follow‐up of the Child Health and Development Studies pregnancy cohort. Circulation. 2015;132:1234–1242. 26391409 10.1161/CIRCULATIONAHA.113.003901 PMC6938224

[6] American College of O, Gynecologists, Task Force on Hypertension in P . Hypertension in pregnancy. Report of the American College of Obstetricians and Gynecologists’ Task Force on Hypertension in Pregnancy. Obstet Gynecol. 2013;122:1122–1131. 24150027 10.1097/01.AOG.0000437382.03963.88

[7] Hauth JC , Ewell MG , Levine RJ , Esterlitz JR , Sibai B , Curet LB , Catalano PM , Morris CD . Pregnancy outcomes in healthy nulliparas who developed hypertension. Calcium for Preeclampsia Prevention Study Group. Obstet Gynecol. 2000;95:24–28. 10636496 10.1016/s0029-7844(99)00462-7

[8] Buchbinder A , Sibai BM , Caritis S , Macpherson C , Hauth J , Lindheimer MD , Klebanoff M , Vandorsten P , Landon M , Paul R , Miodovnik M , Meis P , Thurnau G ; National Institute of Child H, Human Development Network of Maternal‐Fetal Medicine U . Adverse perinatal outcomes are significantly higher in severe gestational hypertension than in mild preeclampsia. Am J Obstet Gynecol. 2002;186:66–71. 11810087 10.1067/mob.2002.120080

[9] Yoder SR , Thornburg LL , Bisognano JD . Hypertension in pregnancy and women of childbearing age. Am J Med. 2009;122:890–895. 19786154 10.1016/j.amjmed.2009.03.036

[10] Tranquilli AL , Dekker G , Magee L , Roberts J , Sibai BM , Steyn W , Zeeman GG , Brown MA . The classification, diagnosis and management of the hypertensive disorders of pregnancy: a revised statement from the ISSHP. Pregnancy Hypertens. 2014;4:97–104. 26104417 10.1016/j.preghy.2014.02.001

[11] Report of the National High Blood Pressure Education Program Working Group on High Blood Pressure in Pregnancy. Am J Obstet Gynecol. 2000;183:S1–S22. 10920346

[12] Melamed N , Ray JG , Hladunewich M , Cox B , Kingdom JC . Gestational hypertension and preeclampsia: are they the same disease? J Obstet Gynaecol Can. 2014;36:642–647. 25184984 10.1016/S1701-2163(15)30545-4

[13] Egeland GM , Klungsoyr K , Oyen N , Tell GS , Naess O , Skjaerven R . Preconception cardiovascular risk factor differences between gestational hypertension and preeclampsia: cohort Norway study. Hypertension. 2016;67:1173–1180. 27113053 10.1161/HYPERTENSIONAHA.116.07099 PMC4861703

[14] Shen M , Smith GN , Rodger M , White RR , Walker MC , Wen SW . Comparison of risk factors and outcomes of gestational hypertension and pre‐eclampsia. PLoS One. 2017;12:e0175914. 28437461 10.1371/journal.pone.0175914 PMC5402970

[15] Hernandez‐Diaz S , Toh S , Cnattingius S . Risk of pre‐eclampsia in first and subsequent pregnancies: prospective cohort study. BMJ. 2009;338:b2255. 19541696 10.1136/bmj.b2255 PMC3269902

[16] Tangeras LH , Austdal M , Skrastad RB , Salvesen KA , Austgulen R , Bathen TF , Iversen AC . Distinct first trimester cytokine profiles for gestational hypertension and preeclampsia. Arterioscler Thromb Vasc Biol. 2015;35:2478–2485. 26404486 10.1161/ATVBAHA.115.305817

[17] Granger JP , Alexander BT , Llinas MT , Bennett WA , Khalil RA . Pathophysiology of hypertension during preeclampsia linking placental ischemia with endothelial dysfunction. Hypertension. 2001;38:718–722. 11566964 10.1161/01.hyp.38.3.718

[18] Powe CE , Levine RJ , Karumanchi SA . Preeclampsia, a disease of the maternal endothelium: the role of antiangiogenic factors and implications for later cardiovascular disease. Circulation. 2011;123:2856–2869. 21690502 10.1161/CIRCULATIONAHA.109.853127 PMC3148781

[19] Lykke JA , Langhoff‐Roos J , Sibai BM , Funai EF , Triche EW , Paidas MJ . Hypertensive pregnancy disorders and subsequent cardiovascular morbidity and type 2 diabetes mellitus in the mother. Hypertension. 2009;53:944–951. 19433776 10.1161/HYPERTENSIONAHA.109.130765

[20] Wikstrom AK , Haglund B , Olovsson M , Lindeberg SN . The risk of maternal ischaemic heart disease after gestational hypertensive disease. BJOG. 2005;112:1486–1491. 16225567 10.1111/j.1471-0528.2005.00733.x

[21] Smith GC , Pell JP , Walsh D . Pregnancy complications and maternal risk of ischaemic heart disease: a retrospective cohort study of 129,290 births. Lancet. 2001;357:2002–2006. 11438131 10.1016/S0140-6736(00)05112-6

[22] Mongraw‐Chaffin ML , Cirillo PM , Cohn BA . Preeclampsia and cardiovascular disease death: prospective evidence from the child health and development studies cohort. Hypertension. 2010;56:166–171. 20516394 10.1161/HYPERTENSIONAHA.110.150078 PMC3037281

[23] Lykke JA , Langhoff‐Roos J , Lockwood CJ , Triche EW , Paidas MJ . Mortality of mothers from cardiovascular and non‐cardiovascular causes following pregnancy complications in first delivery. Paediatr Perinat Epidemiol. 2010;24:323–330. 20618721 10.1111/j.1365-3016.2010.01120.x

[24] Ray JG , Vermeulen MJ , Schull MJ , Redelmeier DA . Cardiovascular health after maternal placental syndromes (CHAMPS): population‐based retrospective cohort study. Lancet. 2005;366:1797–1803. 16298217 10.1016/S0140-6736(05)67726-4

[25] Jonsdottir LS , Arngrimsson R , Geirsson RT , Sigvaldason H , Sigfusson N . Death rates from ischemic heart disease in women with a history of hypertension in pregnancy. Acta Obstet Gynecol Scand. 1995;74:772–776. 8533558 10.3109/00016349509021195

[26] Wilson BJ , Watson MS , Prescott GJ , Sunderland S , Campbell DM , Hannaford P , Smith WC . Hypertensive diseases of pregnancy and risk of hypertension and stroke in later life: results from cohort study. BMJ. 2003;326:845–849. 12702615 10.1136/bmj.326.7394.845 PMC153466

[27] Kestenbaum B , Seliger SL , Easterling TR , Gillen DL , Critchlow CW , Stehman‐Breen CO , Schwartz SM . Cardiovascular and thromboembolic events following hypertensive pregnancy. Am J Kidney Dis. 2003;42:982–989. 14582042 10.1016/j.ajkd.2003.07.001

[28] Cain MA , Salemi JL , Tanner JP , Kirby RS , Salihu HM , Louis JM . Pregnancy as a window to future health: maternal placental syndromes and short‐term cardiovascular outcomes. Am J Obstet Gynecol. 2016;215:484.e1–484.e14. 27263996 10.1016/j.ajog.2016.05.047

[29] Behrens I , Basit S , Melbye M , Lykke JA , Wohlfahrt J , Bundgaard H , Thilaganathan B , Boyd HA . Risk of post‐pregnancy hypertension in women with a history of hypertensive disorders of pregnancy: nationwide cohort study. BMJ. 2017;358:j3078. 28701333 10.1136/bmj.j3078 PMC5506851

[30] Tooher J , Thornton C , Makris A , Ogle R , Korda A , Hennessy A . All hypertensive disorders of pregnancy increase the risk of future cardiovascular disease. Hypertension. 2017;70:798–803. 28893895 10.1161/HYPERTENSIONAHA.117.09246

[31] Irgens LM . The Medical Birth Registry of Norway. Epidemiological research and surveillance throughout 30 years. Acta Obstet Gynecol Scand. 2000;79:435–439. 10857866

[32] Skjaerven R , Gjessing HK , Bakketeig LS . Birthweight by gestational age in Norway. Acta Obstet Gynecol Scand. 2000;79:440–449. 10857867

[33] Irgens HU , Reisaeter L , Irgens LM , Lie RT . Long term mortality of mothers and fathers after pre‐eclampsia: population based cohort study. BMJ. 2001;323:1213–1217. 11719411 10.1136/bmj.323.7323.1213 PMC59993

[34] Ahmed R , Dunford J , Mehran R , Robson S , Kunadian V . Pre‐eclampsia and future cardiovascular risk among women: a review. J Am Coll Cardiol. 2014;63:1815–1822. 24613324 10.1016/j.jacc.2014.02.529

[35] Klungsoyr K , Harmon QE , Skard LB , Simonsen I , Austvoll ET , Alsaker ER , Starling A , Trogstad L , Magnus P , Engel SM . Validity of pre‐eclampsia registration in the medical birth registry of Norway for women participating in the Norwegian mother and child cohort study, 1999–2010. Paediatr Perinat Epidemiol. 2014;28:362–371. 25040774 10.1111/ppe.12138 PMC4167249

[36] Thomsen LC , Klungsoyr K , Roten LT , Tappert C , Araya E , Baerheim G , Tollaksen K , Fenstad MH , Macsali F , Austgulen R , Bjorge L . Validity of the diagnosis of pre‐eclampsia in the Medical Birth Registry of Norway. Acta Obstet Gynecol Scand. 2013;92:943–950. 23621424 10.1111/aogs.12159

[37] Moth FN , Sebastian TR , Horn J , Rich‐Edwards J , Romundstad PR , Asvold BO . Validity of a selection of pregnancy complications in the Medical Birth Registry of Norway. Acta Obstet Gynecol Scand. 2016;95:519–527. 26867143 10.1111/aogs.12868

[38] Dayan N , Lanes A , Walker MC , Spitzer KA , Laskin CA . Effect of chronic hypertension on assisted pregnancy outcomes: a population‐based study in Ontario, Canada. Fertil Steril. 2016;105:1003–1009. 26690007 10.1016/j.fertnstert.2015.11.039

