# Supplementary material for: Association Between Gestational Hypertension and Risk of Cardiovascular Disease Among 617 589 Norwegian Women
Source: J Am Heart Assoc. 2018 May 13;7(10):e008337. doi: 10.1161/JAHA.117.008337 (PMC6015305; doi:10.1161/JAHA.117.008337)
Supplement: Supplementary file 1 — Table S1. HRs and 95% CIs Using Time‐Dependent Covariates for Associations Between GH in at Least 1 Pregnancy During 1980–2009 and Risk of CVD Among 577 185 Women [file JAH3-7-e008337-s001.pdf]

Supplemental Material

Table S1. Hazard ratios (HRs) and 95% confidence intervals (CI) using time-dependent covariates for associations between gestational hypertension (GH) in at least one pregnancy during 1980-2009 and risk of cardiovascular disease (CVD) among 577,185 women.

		No. /events	Unadjusted* HR (95 % CI)	Model 1* † HR (95 % CI)		No./events	Model 2‡ HR (95 % CI)
CVD							
	No GH	526,213/16,257	1 (ref.)	1 (ref.)		560,305/18,491	1 (ref.)
	GH in at least one pregnancy	14,259/871	2.0 (1.9-2.2)	2.0 (1.9-2.2)		16,880/1133	1.9 (1.8-2.0)
CHD							
	No GH	526,213/2148	1 (ref.)	1 (ref.)		560,305/2411	1 (ref.)
	GH in at least one pregnancy	14,259/108	1.7 (1.4-2.1)	1.7 (1.4-2.1)		16,880/143	1.7 (1.4-2.0)
Cerebrovascular disease							
	No GH	526,213/2038	1 (ref.)	1 (ref.)		560,305/2283	1 (ref.)
	GH in at least one pregnancy	14,259/83	1.5 (1.2-1.9)	1.6 (1.3-1.9)		16,880/108	1.5 (1.2-1.8)

GH indicates gestational hypertension; PE, preeclampsia; CVD, cardiovascular disease; CHD, coronary heart disease; HR, hazard ratio; CI, confidence interval.

Additional analyses on all women was performed using information on hypertensive pregnancy disorders in any of the women`s pregnancies (up to five pregnancies). Cox proportional-hazard regression analyses were performed with GH as a time-dependent covariate. Follow-up starts at mother`s age at first delivery (baseline) and mother`s age was applied as the time scale. From the study population of 678, 957 women we did exclusions according to: previous diagnosis of CVD at baseline (n=6385), multiple gestation (n=21,125), births before 20 weeks of gestation/or missing on length of pregnancy (n=69,198), missing on education (n=5058) and women with negative follow–up time, probably due to an erroneous date of death (n=6). We were then left with 577,185 women for these analyses.

*Women with PE (n=36,713) are excluded from the analysis.

†Adjusted for educational level, marital status, birth year of first child and parity (time-dependent) (all are significant in full model).

‡ Women with PE are included in the analysis. Adjusted for educational level, marital status, birth year of first child, parity (time-dependent) and PE (time-dependent) (all are significant in full model).
